# Effects of co-exposure to atrazine and ethanol on the oxidative damage of kidney and liver in Wistar rats

**DOI:** 10.1080/0886022X.2017.1351373

**Published:** 2017-07-25

**Authors:** Sunny O. Abarikwu, Queen C. Duru, Rex-Clovis C. Njoku, Benjamin A. Amadi, Aseme Tamunoibuomie, Enebimoere Keboh

**Affiliations:** Department of Biochemistry, University of Port Harcourt, Choba, Nigeria

**Keywords:** Atrazine, ethanol, malondialdehyde, histopathological damage, kidney, liver, curcumin

## Abstract

Both ethanol (EtoH) and atrazine (ATZ) have hepatic and nephro-toxic effects in rats. In the present study, the toxicity of EtoH (5 g kg^−1^) on the kidney and liver in the absence or in the presence of different doses of ATZ (50, 100, 300 mg kg^−1^) was evaluated after 21 days in rats. Results showed that the mixture effects on catalase and superoxide dismutase activities were more severe in both tissues compared to EtoH alone, especially as the dose of ATZ was increased. Hepatic malondialdehyde level (an index of lipid peroxidation) was increased from 20.32% in the EtoH +50 mg kg^−1^ ATZ-treated rats to 34% in the EtoH +300 mg kg^−1^ ATZ-treated rats compared to the EtoH values. Renal malondialdehyde values remain as high as 81% in the EtoH-treated rats and the different combine exposure groups. Furthermore, as the dose of ATZ in the mixture was increased, serum uric acid level increased compared to the EtoH values. When the EtoH +300 mg kg^−1^ ATZ-animals were pretreated with curcumin (an antioxidant), the histopathological changes and peroxidative damages in both tissues were blocked. The exposure of EtoH-treated rats to ATZ enhanced renal and hepatic peroxidative damages in rats.

## Introduction

Atrazine (ATZ) has been widely used as herbicide in most parts of the world, but its use is declining in Europe because of its potentially unacceptable effects on groundwater, demonstrated harmful effects on wildlife, and potential health hazards for humans [1,2]. In addition to industrial sources, ATZ is present in the environment as surface and ground water contaminant [3,4]. Other routes of exposure to triazine include dermal contact, and inhalation from occupational sources [5]. Because ATZ accumulates in brain, gall bladder, liver and gut of some fishes, the consumption of contaminated fishes can also contribute to human exposure [6]. Triazine herbicides (e.g. simazine) have also been detected in the urine of pregnant women at a median concentration of 1 mg/L [7].

An association between environmental and occupational exposure to ATZ and health effects, such as ovarian cancer, breast cancer and tumors of the mammary gland, DNA damage, endocrine disruption, developmental delays and abnormalities, decreased semen quality and birth defects have been reported in studies with several experimental models, e.g. human, rodent cells, amphibian and mammalian cells [8–13]. Other studies also reported that ATZ induces oxidative damage, cytotoxicity and apoptosis in several *in vivo* and *in vitro* models systems [14–20]. The neurotoxic [19,21,22], hepatotoxic [16,23,24], and nephrotoxic effects [25,26] of triazine herbicides (ATZ) in both *in vivo* and *in vitro* experimental models are also well known.

Ethanol (EtoH) is consumed worldwide in tremendous amounts, and in the form of alcoholic beverages, it is considered as an integral part of food supply in most countries. Some heavy alcohol users, progress to having alcoholic hepatitis that associates with significant mortality [27]. Kidney filtration is also affected by chronic ethanol use [28], and the mortality of patients hospitalized with alcoholic hepatitis correlates with the rapid development of kidney dysfunction, not the underlying hepatitis [27,29]. In animal models (e.g. rats), exposure to EtoH can lead to liver and kidney injury [30–32]. Previous studies have shown that EtoH produces oxidative stress in most organisms, and with the formation of lipid peroxides and free radicals linked to its adverse health effects [33,34]. Furthermore, EtoH exposure can influence the toxicity of various environmental chemicals by altering the expression or activity of xenobiotics-metabolizing enzymes [33,34] and the formation of free radicals and reactive oxygen species [34]. Simultaneous exposures to environmental chemicals in the natural as well as in the occupational setting mimic real human scenario, and individual effects of chemicals have been reported to be significantly influenced by mixture interactions [35]. The liver is usually the first target of ingested compounds before they get into the body fluids and thus exposed to high concentrations of these chemicals [36]. This intense role of the kidney in the processing of foreign chemicals and homeostasis makes it vulnerable to the adverse effects of xenobiotics and reactive metabolites-induced toxicity [36]. Therefore, whenever xenobiotics that are metabolize in the liver and processed in the kidney, are taken by an individual who is also chronically consuming EtoH, the combined effects of these agents on the hepatic and renal health has to be considered [37,38]. Therefore, the objective of the present study was to evaluate whether repeated co-exposures to EtoH and the triazine herbicides (e.g. ATZ) might influence *in vivo* the oxidative status and histopathological changes in the liver and kidney of male Wistar rats.

## Materials and methods

### Animals and experimental design

Thirty male Wistar rats (4–5 weeks of age, 78–90 g) were provided by the Animal House of the Department of Biochemistry, University of Port Harcourt, and were randomly assigned to five (5) groups of six (6) animals per group. Rats were allowed to acclimatize for 1 week prior to the start of study. The animals were maintained under 12-h light:12-h dark cycles and were supplied with drinking water and fed *ad libitum*. International rules and regulations guiding the handling and care of animals were followed throughout the study. The EtoH group was orally administered EtoH by gavage, 5 g/kg (50% v/v) body weight at a constant volume of 2 mL/kg body weight of corn oil (vehicle) three times a week for 21 days. Different doses of ATZ (50, 100, 300 mg/kg body weight) were also prepared in the vehicle, corn oil and administered to the EtoH group at 2 mL/kg body weight, three times a week for 21 days. The control rats were administered with equivalent volumes of corn oil only (2 mL/kg body weight). The doses of ATZ and treatment period were based on the LD50 (LD50/7; for an oral dose) and our previous studies [39], respectively. The dose of EtoH was based on previous studies that reported changes in the antioxidative system of the kidney and liver of rats [38]. In another experiment, the EtoH- **(**5 g/kg; 50% v/v) and ATZ- (300 mg/kg) treated animals were co-administered with curcumin (100 mg/kg/day) [40] simultaneously three times a week for 21 days. At the end of 21 days, the animals were fasted overnight, weighed and killed by cervical dislocation without anesthesia. Body weight was recorded prior to sacrifice. Blood samples were collected for serum marker analysis. The paired kidney and liver were dissected out quickly and washed in 1.15% KCl (ice cold) and pat-dried and the wet weight taken. The tissues were homogenized in ice-cold 0.1 M Tris–HCl buffer (pH 7.4) to produce 10% homogenate. The homogenate was centrifuged at 5000*g* and 4 °C for 15 min and the supernatant was separated to measure the biochemical parameters of oxidative stress.

## Oxidative stress assay

### Malondialdehyde measurement

Lipid peroxidation of hepatic and renal tissues of all groups were measured as thiobarbituric acid reactive substances according to the method described previously [41]. Briefly, 0.5 mL aliquots of the homogenate was mixed thoroughly with 0.5 mL of 20% trichloroacetic acid and subjected to centrifugation at 3000*g*. Equal volume of 0.67% thiobarbituric acid (dissolved in 0.1 M HCl solution) was mixed with the supernatant; then the mixture was heated at 100 °C for 1 h. After cooling with tap water, the absorbance of the pink colored solution was then measured at 532 nm using 1,1,3,3-tetraethoxypropane as a standard. The level of MDA was expressed as micromoles per milligram of protein.

### Reduced glutathione and glutathione peroxidase measurements

Reduced glutathione (GSH) was determined in the liver and kidney homogenates according to the method described by Sedlak and Lindsay [42]. Briefly, aliquots (0.5 mL) of the kidney and liver homogenates were deproteinized with 10% TCA, centrifuged at 3000*g* for 10 min and the supernatant collected. The reaction mixture containing 0.5 mL of the sample supernatant, 4 mL phosphate buffer (0.1 M, pH 7.4) and 0.5 mL Ellman’s reagent, 5,5′-dithiobis 2-nitro benzoic acid (0.4% in 0.1 M, pH 7.4 phosphate buffer) were mixed and incubated in the dark at room temperature for 5 min. The yellow color that developed was read immediately at 412 nm using GSH as a standard. The results were expressed as microgram per milligram protein. The activity of glutathione peroxidase (GSH-Px) was determined by the method of Rotruck et al. [43] Briefly, the assay mixture containing 0.5 mL of sodium phosphate buffer, 0.1 mL of 10 mM sodium azide, 0.2 mL of 4 mM reduced glutathione, 0.1 mL of 2.5 mM H_2_O_2_ and 0.5 mL sample supernatant was taken and the total volume was made up to 2.0 mL with distilled water. The tubes were incubated at 37 °C for 3 min and the reaction was terminated by the addition of 0.5 mL 10% TCA. To determine the residual glutathione content, the supernatant was removed after centrifugation and to this 4.0 mL of disodium hydrogen phosphate (0.3 M) solution and 1 mL of the Ellman’s reagent were added. The color developed was read at 412 nm against a reagent blank containing only phosphate solution and Ellman’s reagent on a spectrophotometer. Suitable aliquots of the standard were also treated similarly. The enzyme activity was expressed as units per milligram of protein.

### Assay of superoxide dismutase and catalase activities

The activity of superoxide dismutase (SOD) was determined by the method of Misra and Fridovich [44]. The reaction was started by the addition of 0.3 mL of freshly prepared epinephrine (0.01%) to the mixture containing 2.5 mL carbonate buffer (0.05 M, pH 10.2) and 0.2 mL sample. The mixture was quickly mixed by inversion and immediately read at 480 nm against blank containing all the components except the sample at 30 s interval for 3 min in a spectrophotometer. Catalase activity was determined according to the method given by Clairborne [45] with slight modifications. Briefly, 0.2 mL of sample (equivalent to 20–50 mg protein) was added to (0.7 mL) 50 mM of phosphate buffer (pH 7.4) containing (0.1 mL) 100 mM of H_2_O_2_ in a total of 1 mL. The reaction mixture was incubated for 2 min at 37 °C and the rate of absorbance change at 240 nm was recorded, which indicated the decomposition of H_2_O_2_. Activities were calculated using the molar extinction coefficient of H_2_O_2_ at 240 nm, 43.59 L/mol cm. One unit of catalase activity equals the amount of protein that converts 1 mmol H_2_O_2_/min. Tissue protein was measured spectrophotometrically according to the method by Lowry et al. [46].

### Serum uric acid measurement

Uric acid level was estimated in the serum using Randox commercial kits (RANDOX Laboratories Ltd., Crumlin, UK), following strictly the instructions provided by the manufacturers.

### Histopathological examination of liver and kidney

At necropsy, one kidney and a portion of the liver of each rat was fixed in 10% formalin. After 72 h, formalin-fixed tissues were embedded in paraffin wax according to the routine procedure, and 5-μm-thick sections were cut with a rotary microtome. The sections were stained with hematoxylin and eosin (H & E). Histological specimens were examined in light microscopy (Olympus CX31; Olympus Co., Tokyo, Japan).

### Statistics

All data were presented as mean ± SD. The variance among the experimental groups was compared by one-way ANOVA, with the *post-hoc* Tukey test. Differences were considered significant when *p* < .05. The statistical analyses were performed by GraphPad InStat, version3.01 (GraphPad, San Diego, CA). 

## Results

### Body and organs weight

The body weight, liver and kidney weights are shown in Table 1. The animals that received 300 mg/kg ATZ plus EtoH for 21 days had their absolute kidney and liver weights statistically decreased by 11.58 and 26.7%, respectively. However, these significant differences were not found when the absolute liver and kidney weights were converted to relative weights and compared to the control animals or EtoH-treated rats. The final body weight of the EtoH +300 mg/kg ATZ animals was statistically decreased compared to the control or EtoH alone treated animals.

**Table 1.** Body weights, liver and kidney weights of animals at the end of experiment.

	Liver		Kidney		Initial	Final
Groups	Absolute (g)	Relative (g)	Absolute (g)	Relative (g)	Body weights (g)	
Control	4.65 ± 0.49	3.18 ± 0.62	0.95 ± 0.01	0.66 ± 0.04	80.16 ± 5.67	142.94 ± 9.26
EtoH	4.59 ± 0.53	3.29 ± 0.38	0.9 ± 0.09	0.64 ± 0.07	77.7 ± 13.21	139.78 ± 7.16
EtoH +50 mg/kg ATZ	4.21 ± 0.63	3.18 ± 0.48	0.91 ± 0.08	0.69 ± 0.06	84.16 ± 11.79	132.54 ± 19.1
EtoH +100 mg/kg ATZ	4.25 ± 0.47	3.06 ± 0.34	0.94 ± 0.14	0.68 ± 0.1	76.42 ± 7.41	139.1 ± 18.38
EtoH +300 mg/kg ATZ	3.41 ± 0.41*	2.89 ± 0.35	0.84 ± 0.05*	0.71 ± 0.04	89.64 ± 21.67	117.72 ± 26.48*

Data are presented as the mean ± SD (*n* = 6).

* Significantly different from the control group (*p* < .05).

### Malondialdehyde and reduced glutathione concentrations and glutathione peroxidase assay

**Figure 1.**
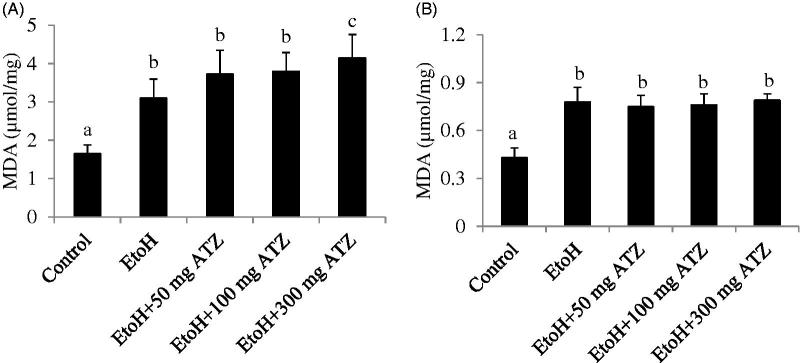
Effects of ethanol (EtoH) and the combination with different doses of atrazine (ATZ) on malondialdehyde (MDA) concentration in the liver (A) and kidney (B) of rats after 3 weeks treatment regimen. Data are presented as the mean ± SD (*n* = 6). Data with different superscripts are significantly different (*p* < .05).

In the liver, EtoH exposure induced an increase in MDA level compared to the control animals. The EtoH- and ATZ-treated animals showed significant increase in MDA level when compared to the EtoH animals as the dose of ATZ increases (*p* < .05, Figure 1(A)). In the kidney, the level of MDA was higher in the EtoH-treated animals in relation to the control group (*p* < .05). The combine exposure groups also showed significant increase in MDA level but there were no dose-dependent increases in the MDA concentration among the combine exposure groups or in relation to the EtoH group (*p* > .05, Figure 1(B)). The GSH concentration was decreased by 22% in the EtoH +100 mg/kg ATZ or EtoH +300 mg/kg ATZ animals in relation to the control group or EtoH-treated animals (*p* < .05, Table 2). The GSH-Px assay in the kidney showed a significant increase in the EtoH-treated animals compared to the control animals (*p* < .05, Table 1). The EtoH +50 mg/kg ATZ and EtoH +100 mg/kg ATZ groups also showed increase in GSH-Px activity but were not different in relation to the EtoH group. However, the GSH-Px activity in the EtoH +300 mg/kg ATZ animals was higher by 17% when compared to the EtoH-treated animals (*p* < .05, Table 2).

**Table 2.** Effects of ethanol (EtoH) and the combination with different doses of atrazine (ATZ) on catalase (CAT), superoxide dismutase (SOD), reduced glutathione (GSH) and glutathione peroxidase (GSH-px) activities in the liver and kidney at the end of experiment.

	Liver				Kidney			
Group	CAT	SOD	GSH	GSH-Px	CAT	SOD	GSH	GSH-Px
Control	4.22 ± 2.51	171.1 ± 31.96	82.03 ± 11.87	27.83 ± 2.15	3.18 ± 1.22	311.8 ± 20.41	21.68 ± 1.39	6.15 ± 0.31
EtoH	15.68 ± 4.74*	452.78 ± 44.51*	87.48 ± 15.13	26.36 ± 2.98	1.24 ± 0.1*	345.9 ± 21.49	21.66 ± 1.32	8.4 ± 0.49*
EtoH +50 mg/kg ATZ	16.55 ± 4.02*	410.56 ± 26.72*	89.99 ± 15.64	29.3 ± 4.17	1.09 ± 0.3*	141.7 ± 27.24*	20.35 ± 6.68	7.57 ± 0.57*
EtoH +100 mg/kg ATZ	23.73 ± 9.24*	581.61 ± 23.26†	86.22 ± 11.1	28.95 ± 1.14	0.31 ± 0.05†	106.9 ± 20.85*	16.98 ± 1.6*,†	7.72 ± 0.98*
EtoH +300 mg/kg ATZ	34.61 ± 2.77†	677.76 ± 79.74†	88.64 ± 13.4	34.27 ± 6.32	0.26 ± 0.06†	48.22 ± 16.39*,†	16.27 ± 1.52*,†	9.8 ± 0.4*,†

Data are presented as the mean ± SD (*n* = 6).

* Significantly different from the control group.

† Significantly different from the EtoH group (*p* < .05). CAT (Units/mg), SOD (nmoles epinephrine oxidized/min/mg protein), GSH (μg GSH/mg), GSH-Px (μg residual GSH remaining/min/mg).

### Catalase and superoxide dismutase assays

In the liver, EtoH exposure increased CAT activity by 271.56% compared to the control group. The activity of CAT was further increased by 51.34 and 462.32% in the EtoH +100 mg/kg ATZ combine exposure group when compared to EtoH and control groups, respectively. When EtoH was co-administered with the 300 mg/kg ATZ, CAT activity was further increased by 120.73 and 720% when compared to the EtoH and control groups, respectively (*p* < .05, Table 2). The SOD assay also showed significant increase (164.63%) in the liver of EtoH-treated animals when compared to the control group. The activity of SOD was increased from 28.45% in the EtoH +100 mg/kg ATZ to 49.69% in the EtoH +300 mg/kg ATZ compared to the EtoH group, and from 240% in the EtoH +100 mg/kg ATZ to 296.12% in the EtoH +300 mg/kg ATZ compared to the control group (*p* < .05, Table 2).

### The role of oxidative stress in the combine effects of EtoH and ATZ in the liver and kidney

**Figure 2.**
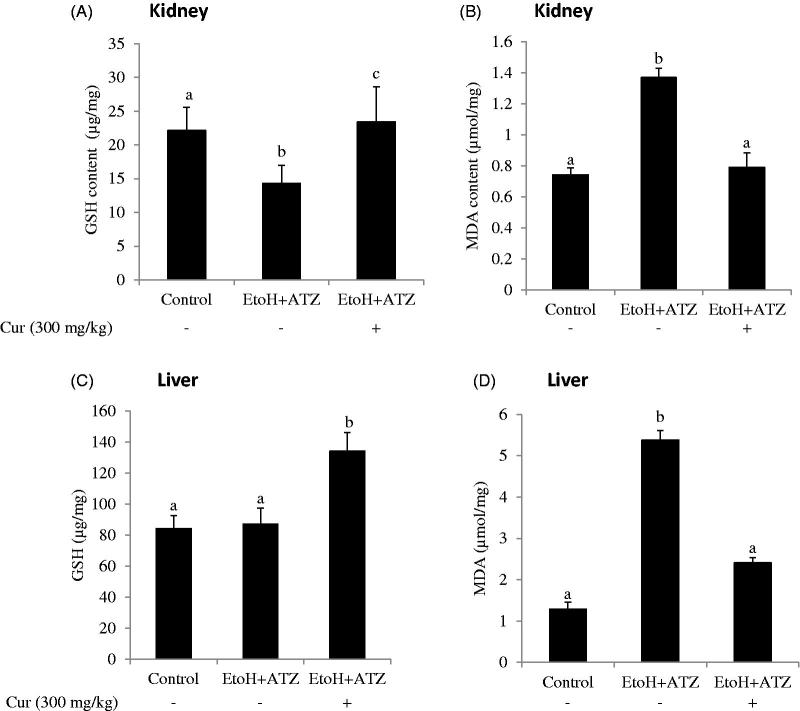
The role of oxidative stress in both atrazine (ATZ) + ethanol (EtoH) induced hepato-renal toxicity in rats. Data are presented as the mean ± SD (*n* = 6). *Values with different superscripts are significantly different (*p* < .05).

The co-administration of curcumin (Cur) increased the GSH level and decreased the MDA concentration observed in the kidney of the combined exposure group (EtoH +300 mg/kg ATZ). Similarly, the increased hepatic MDA level induced by the mixture effect of ATZ and EtoH was prevented by curcumin. Furthermore, curcumin co-administration increased hepatic GSH level by 59.1% in relation to the control value whereas no change in hepatic GSH level was observed in the combined exposure group (Figure 2).

### Serum uric acid level in EtoH and ATZ-treated rats

**Figure 3.**
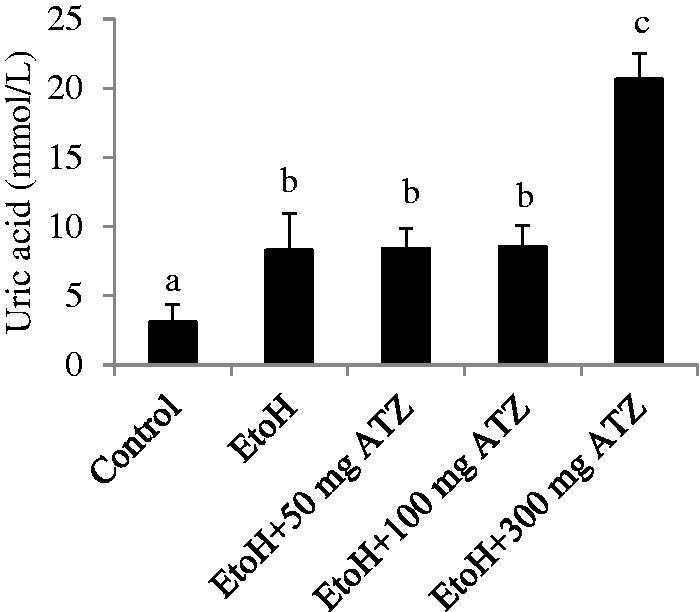
Effects of ethanol (EtoH) and the combination with different doses of atrazine (ATZ) on serum uric acid level. Data are presented as the mean ± SD (*n* = 6). *Values with different superscripts are significantly different (*p* < .05).

The serum level of uric acid was increased by 167.52% as a result of EtoH administration (*p* < .05). The EtoH-exposed animals that were treated with 300 mg/kg ATZ have higher levels of serum uric acid (148.44%) when compared to the EtoH-treated animals (Figure 3, *p* < .05).

### Histological findings

**Figure 4.**
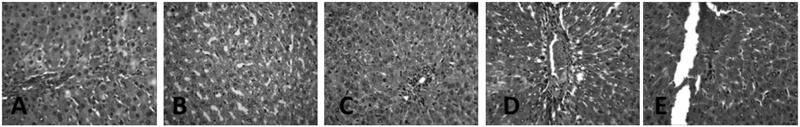
Histopathology sections of the liver of ATZ- and EtoH-treated rats. (A) Control: No lesion seen. (B) EtoH: There is a very mild diffuse hydropic degeneration of hepatocytes. (C) EtoH +50 ATZ: There is a very mild periportal cellular infiltration by mononuclear cells. (D) EtoH +100 ATZ: There is a mild portal congestion, with mild periportal cellular infiltration. (E) EtoH +300 ATZ: There is a mild portal and central venous congestion. H & E, ×400 Mag.

**Figure 5.**
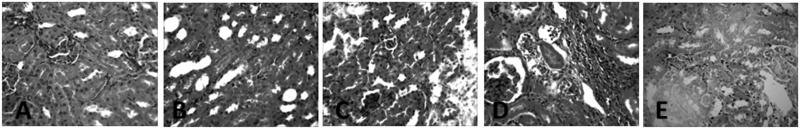
Histopathology of the kidney section of ATZ- and EtoH-treated rats. (A) Control: No visible lesions seen. (B) EtoH: No visible lesion seen. (C) EtoH +50 ATZ: There is a very mild renal cortical congestion. (D) EtoH +100 ATZ: The periglomerular interstitium appears infiltrated. There is a mild congestion of the renal cortex. (E) EtoH +300 ATZ: The renal tubules appear degenerate; many have proteinaceous casts in their lumina. H & E, ×400 Mag.

**Figure 6.**
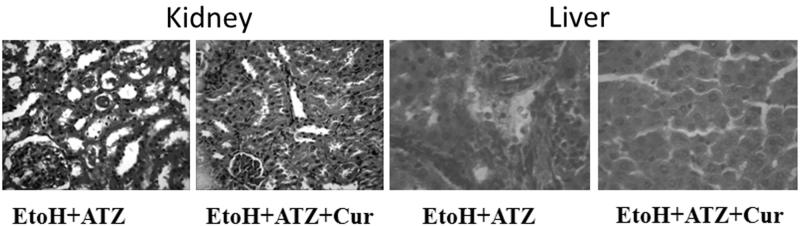
Protective effects of curcumin (Cur) on the histopathological changes in the kidney and liver induced by the combine effects of EtoH (5 mg/kg, 50%v/v) plus ATZ (300 mg/kg b.wt.) in rats. In the kidney, many tubules are degenerate and contain protein casts in the lumina of EtoH + ATZ animals whereas no visible lesions seen in the kidney of EtoH + ATZ co-administered Cur animals. H & E, ×400 Mag. There is a mild to moderate portal congestion and periportal cellular infiltration by mononuclear cells in the EtoH + ATZ animals whereas no visible lesions seen in the liver of EtoH + ATZ animals co-administered Cur animals.

The liver of the control group showed normal histological architecture and had no visible lesion. Ethanol exposure caused a very mild diffuse hydropic degeneration of hepatocytes. The liver of the EtoH +50 mg/kg ATZ animals showed a very mild periportal cellular infiltration by mononuclear cells. There was also a mild portal congestion, with mild periportal cellular infiltration in the liver of the EtoH +100 mg/kg ATZ-treated animals. Similarly, the liver of the EtoH +300 mg/kg ATZ-exposed animals showed mild portal and central venous congestion (Figure 4). In the kidney, the control group and the EtoH-treated animals showed no visible lesion (Figure 5). The kidney of the EtoH +50 mg/kg ATZ-exposed animals showed very mild renal cortical congestion. Furthermore, the periglomerular interstitium of the EtoH +100 mg/kg ATZ-exposed animals appears infiltrated and have mild congestion of the renal cortex. The renal tubules of the EtoH +300 mg/kg ATZ animals appear to degenerate and many of the tubules have proteinaceous casts in their lumina (Figure 5). Interestingly, all of the histological changes in the kidney and liver of the EtoH +300 mg/kg ATZ animals were attenuated by co-administration of curcumin (Figure 6).

## Discussion

The present study investigated the effects of concomitant EtoH-ATZ co-exposure on the oxidative status of liver and kidney as well as hepato-renal injury in terms of histological changes, and then associated the hepato-renal injury to the oxidative stress status of both organs. For this purpose, the activities of CAT, SOD, GSH-Px and the concentrations of GSH and MDA as indicators of oxidative stress were determined in both the liver and kidney of rats. To achieve this, different doses of ATZ (50, 100, 300 mg/kg) were combined with EtoH (5 g/kg 50%v/v) during the 21 day co-exposure regimen. On the basis of our findings, we wanted to elucidate whether the risk of EtoH-induced oxidative damage in the liver and kidney may be influenced by ATZ. Thus, we used an experimental model of rats’ exposure to ATZ that have been reported to induce oxidative stress in several experimental models [20,26,47]. To further elucidate the role of oxidative stress in the observed hepato-renal injury, curcumin, a natural flavonoid, with established antioxidant properties [40] was used to modulate the potentials of EtoH-ATZ mixtures to induced oxidative stress in the liver and kidney.

The decreased absolute liver and kidney weights in the EtoH +300 mg/kg ATZ animals compared to the EtoH-treated rats could be that these groups of animals had less body weight gain compared with the control group. The results obtained regarding the activities of the antioxidant enzymes, SOD and CAT, and the concentration of MDA (an indicator of lipid peroxidation) in the liver and kidney clearly indicate that EtoH was able to induce oxidative stress during repeated separate administration as well as during co-exposure with ATZ and especially with higher doses of ATZ. Although SOD activity was not changed by EtoH alone in the kidney, the decreased CAT activity in the kidney, which might be due to inhibition caused by excess reactive oxygen species (e.g. H_2_O_2_) was capable of inducing lipid peroxidation in these animals [48]. It therefore seems likely that the decreased CAT activity along with the simultaneous increase in the activity of GSH-Px and MDA concentration as well as the unchanged GSH level and SOD activity in the kidney of EtoH-treated animals reflect the adverse effects of EtoH on this finely balanced antioxidant system.

An important finding in this study is that lipid peroxidation, reflected as MDA concentration in the kidney at the co-exposure to EtoH and different doses of ATZ was not intensified compared to the separate effect of EtoH alone, whereas in the liver, the lipid peroxidation was markedly more advanced in the combined exposure group and especially in the EtoH-treated animals co-exposed to the highest dose of ATZ (300 mg/kg b.wt.) than after EtoH alone. The liver being the main site of EtoH and ATZ biotransformation and target organs for most xenobiotics could be most susceptible to chemically-induced peroxidative damage than the kidney [49].

It was also observed that in the EtoH-applied rats alone, uric acid concentration was significantly increased compared to control values. This increase was higher when the EtoH-alone applied rats were simultaneously co-exposed with the highest dose of ATZ (300 mg/kg). It is known that uric acid is a valuable index of renal function in rats [16,26]. Therefore, the increase in uric acid level observed in the EtoH +300 mg/kg ATZ animals study might indicate the possibility of a more severe effect on renal function.

The results on histological analysis in the present study showed that EtoH consumption induced mild diffuse hydropic degeneration of hepatocytes over a 21-day period. These observations are consistent with several studies in different experimental models of EtoH exposure in rats [30,31], and confirm the pathogenic role of EtoH-induced oxidative stress in the liver. The EtoH-applied animals that were co-exposed to 50, 100 and 300 mg/kg ATZ showed very mild periportal cellular infiltration by mononuclear cells, mild portal congestion, with mild periportal cellular infiltration, and mild portal and central venous congestion, respectively. The kidney of the EtoH-applied rats did not show any perceptible visible lesion, suggesting that the dose of EtoH employed in the present study was insufficient to induce pathological changes in rats in amounts relevant to humans. However, there were mild renal cortical congestions in the EtoH +50 mg/kg ATZ-treated rats. The periglomerular interstitium appears infiltrated with evidence of mild congestion of the renal cortex in the EtoH +100 mg/kg ATZ animals. Finally, in the EtoH +300 mg/kg ATZ animals, the renal tubules appear to degenerate and many have proteinaceous casts in their lumina. Thus, the pathological changes of the kidney observed in the present study could be attributable to ATZ effects alone [26]. Because the changes observed in the obtained results for serum uric acid in the present study were more marked when EtoH administration was used in combination with ATZ and especially with the large dose ATZ (300 mg/kg body weight) than with separate and repeated treatments with EtoH alone, it is speculated that the exposure to both of these chemicals may play a significant role in aggravating certain kidney pathologies, e.g. renal dysfunction and injury associated with an increased level of lipid peroxidation. Hence, antioxidant treatment strategy should prevent the onset of peroxidative damage [50]. Consequently, we tested this hypothesis with curcumin, a natural phenolic compound with well-established antioxidant properties [40], and observed that in the kidney, the decreased GSH and the elevated MDA level in both liver and kidney of EtoH +300 mg/kg ATZ animals were blocked in the presence of curcumin. Furthermore, the associated histopathological changes induced by the combined effects of EtoH and ATZ (300 mg/kg b.wt.) in both kidney and liver were reduced by curcumin.

In conclusion, EtoH–ATZ co-exposure has the potential to impair hepatic and renal function much more than is observed with EtoH treatment alone through the induction of peroxidative damage and histopathological changes. Therefore, the risk of EtoH-induced hepatic and renal damages may be influenced by triazine herbicides (e.g. ATZ), especially at higher doses. These findings have crucial implications for renal and hepatic health especially for humans occupationally exposed to atrazine and consume alcoholic beverages occasionally or habitually.
